# Comprehensive histone epigenetics: A mass spectrometry based screening assay to measure epigenetic toxicity

**DOI:** 10.1016/j.mex.2020.101055

**Published:** 2020-09-05

**Authors:** Sigrid Verhelst, Laura De Clerck, Sander Willems, Bart Van Puyvelde, Simon Daled, Dieter Deforce, Maarten Dhaenens

**Affiliations:** ProGenTomics, Laboratory of Pharmaceutical Biotechnology, Ghent University, Ottergemsesteenweg 460, 9000, Ghent, Belgium

**Keywords:** Histone post-translational modifications, Toxicoepigenetics, Pharmacoepigenetics, LC-MS/MS, Proteomics, Drug safety, AUC, area under the curve, DDA, data-dependent acquisition, DIA, data-independent acquisition, DTT, dithiothreitol, FA, formic acid, FDR, false discovery rate, GABA, gamma-aminobutyric acid, GRX, gingisrex, HAT, histone acetyltransferase, HDACi, histone deacetylase inhibitor, hESC, human embryonic stem cell, HLB, hypotonic lysis buffer, HPLC, high-performance liquid chromatography, hPTM, histone post-translational modification, K, Lysine, M, Methionine, MS, Mass spectrometry, MS/MS, tandem mass spectrometry, N, asparagine, PBS, phosphate buffered saline, Q, glutamine, R, arginine, RA, relative abundance, RP, reversed phase, RT, room temperature, S, serine, SWATH, sequential window acquisition of all theoretical fragment ion spectra, T, threonine, TEAB, triethylammonium bicarbonate, VPA, valproic acid, Y, tyrosine

## Abstract

Evidence of the involvement of epigenetics in pathologies such as cancer, diabetes, and neurodegeneration has increased global interest in epigenetic modifications. For nearly thirty years, it has been known that cancer cells exhibit abnormal DNA methylation patterns. In contrast, the large-scale analysis of histone post-translational modifications (hPTMs) has lagged behind because classically, histone modification analysis has relied on site specific antibody-based techniques. Mass spectrometry (MS) is a technique that holds the promise to picture the histone code comprehensively in a single experiment. Therefore, we developed an MS-based method that is capable of tracking all possible hPTMs in an untargeted approach. In this way, trends in single and combinatorial hPTMs can be reported and enable prediction of the epigenetic toxicity of compounds. Moreover, this method is based on the use of human cells to provide preliminary data, thereby omitting the need to sacrifice laboratory animals. Improving the workflow and the user-friendliness in order to become a high throughput, easily applicable, toxicological screening assay is an ongoing effort. Still, this novel toxicoepigenetic assay and the data it generates holds great potential for, among others, pharmaceutical industry, food science, clinical diagnostics and, environmental toxicity screening.

• There is a growing interest in epigenetic modifications, and more specifically in histone post-translational modifications (hPTMs).
• We describe an MS-based workflow that is capable of tracking all possible hPTMs in an untargeted approach that makes use of human cells.
• Improving the workflow and the user-friendliness in order to become a high throughput, easily applicable, toxicological screening assay is an ongoing effort.

## Graphical abstract

**Figure:**
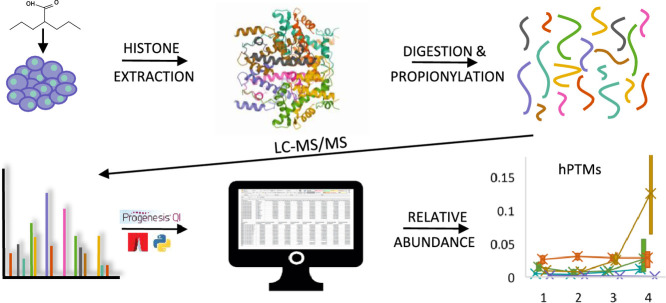

Specifications Table

**Table:** 

Subject Area	Pharmacology, Toxicology and Pharmaceutical Science
More specific subject area	Toxicoepigenetics
Method name	Comprehensive histone epigenetics
Name and reference of original method	OECD guidelines for the Testing of Chemicals, Section 4 OECD (2009), *carcinogenicity studies*, Test Guideline No. 451, OECD Guidelines for the Testing of Chemicals, OECD, Paris. OECD (2009), *combined chronic toxicity\carcinogenicity studies*, Test Guideline No. 453, OECD Guidelines for the Testing of Chemicals, OECD, Paris. OECD (2009), *Prenatal developmental toxicity study*, Test Guideline No. 414, OECD Guidelines for the Testing of Chemicals, OECD, Paris.
Resource availability	Data have been deposited to the ProteomeXchange Consortium via the PRIDE partner repository with the dataset identifier PXD019652 and 10.6019/PXD019652.

## Background

Evidence of the involvement of epigenetics in pathologies such as cancer, diabetes, and neurodegeneration has increased global interest in epigenetic modifications and their impact on the human body [2,3]. For a long time, research focused almost exclusively on two types of epigenetic modifications: DNA methylation and non-coding RNAs [4]. The role of histones in epigenetics, in contrast, has remained largely fragmented, until recently.

**Fig. 1.1:**
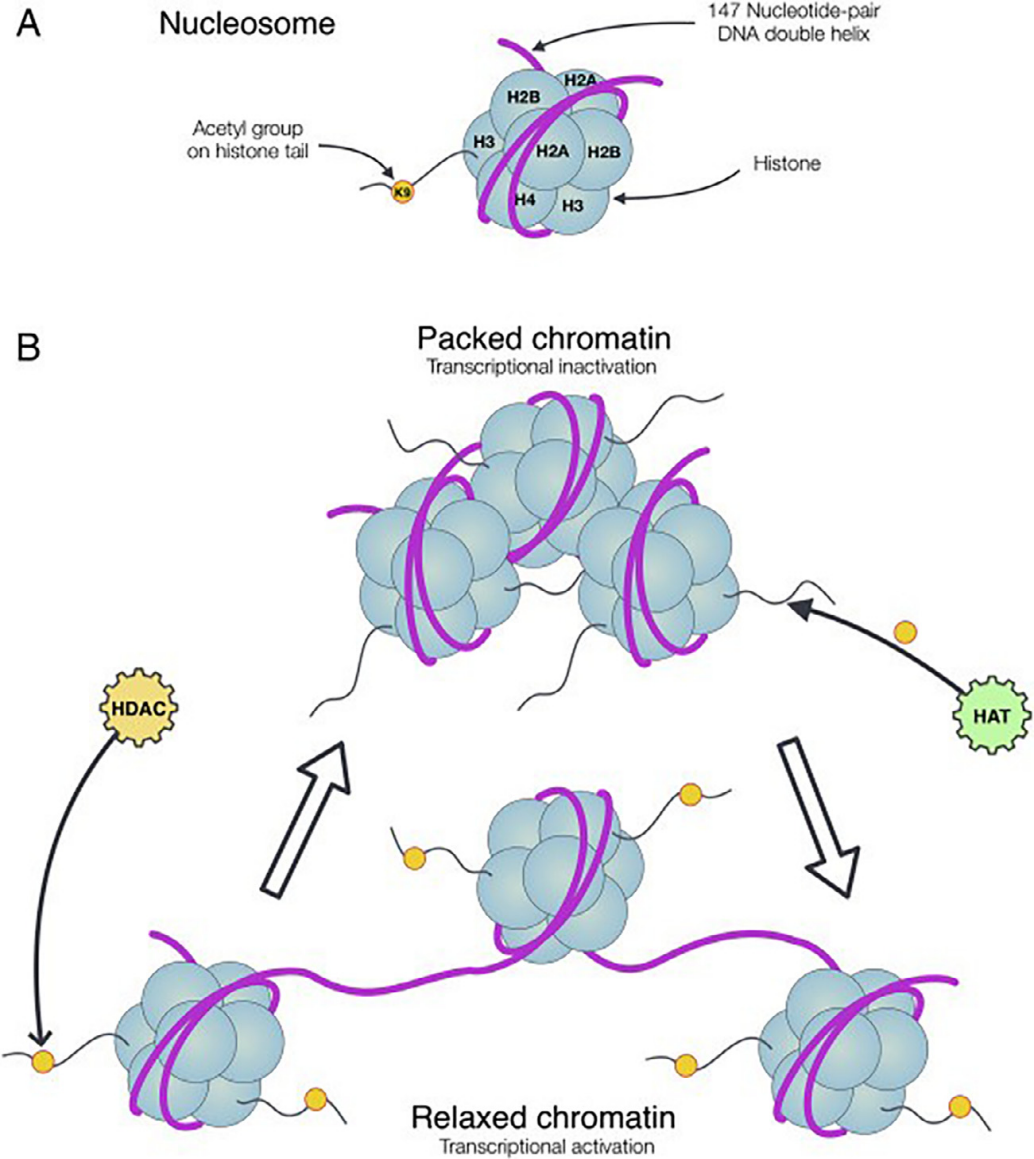
Histones and chromatin structure. (A) Representation of a nucleosome consisting of the core histones (H2A, H2B, H3, and H4) around which the DNA double helix is wrapped. (B) Multiple nucleosomes converge to form chromatin. Chromatin can be either in a packed or in a relaxed state (mediated through e.g. acetylation), respectively, resulting in transcriptional inactivation and activation. Figure adapted from "Histone deacetylases and their roles in mineralized tissue regeneration," by N.C.-N. Huynh et al., 2017, *Bone Reports, 7,* p.34. Copyright 2017 by The Authors [1]**.**

Histones are proteins present in the nucleus of an eukaryotic cell. Approximately 147 base pairs of DNA are wrapped around an octamer of the core histone proteins (i.e. H2A, H2B, H3, and H4), resulting in a nucleosome. The nucleosome in turn is the basic unit of chromatin i.e. the state in which the eukaryotic DNA is packed. Histones typically possess an N-terminal tail that is a template for a plethora of histone post-translational modifications (hPTMs) [4,5] (Fig. 1.1). Over time, a variety of these hPTMs have been discovered, of which acetylation and methylation are the best known. However, new discoveries continue raising the complexity of this already exceedingly complicated biological phenomenon [6].

Epigenetics is defined as ‘*the study of changes in organisms caused by modification of gene expression rather than alteration of the genetic code itself’* [7]. hPTMs are such changes that have an impact on gene expression, without affecting the genetic code. In particular, hPTMs are known to interfere with the histone-DNA interplay. Firstly, the physicochemical properties of hPTMs can affect the charge, leading to a change in interaction with the negatively charged DNA. Subsequently, this potentially results in either a more relaxed or a more packed structure of chromatin, respectively called euchromatin and heterochromatin [4,5] (Fig. 1.1). Secondly, hPTMs can influence the chromatin state either by attracting chromatin-modifying enzymes (writers and erasers) or by binding proteins (readers). These alterations in chromatin state affect transcription efficiency, resulting in the disruption of homeostasis and potentially diseases [5].

Although hPTMs have been studied for decades, new discoveries keep on raising the awareness of the complexity of this biological phenomenon [6,^8^,9]. Especially the modification crosstalk defining the proverbial grammar of histone epigenetics is not well understood. Paradoxically, this is in part due to the success of sequencing-based epigenomic profiling approaches such as ChIP-seq. These approaches rely on antibodies to localize specific hPTMs in the genome. While these methods yield highly informative data about a small number of targeted hPTMs, they cannot be scaled up to study combinations of hPTMs. We recently showed that the lack of such a comprehensive understanding of hPTMs has led to fundamental misconceptions about histone biology [10,11]. Moreover, the context in which hPTMs reside has a major impact on the final biological outcome [12]. It will therefore be crucial to study the dynamics of all hPTMs simultaneously in an untargeted way.

Mass spectrometry (MS) is a prime candidate to perform such comprehensive histone epigenetic profiling [13]. In this context, we developed an MS-based screening method that is capable of tracking all possible hPTMs in an untargeted way. Besides, this method makes use of cells (e.g. human embryonic stem cells or hESCs) to provide preliminary data, thereby omitting the need to sacrifice laboratory animals. This results in an assay that holds promises for, among others, toxicoepigenetics, pharmacoepigenetics, food safety, and environmental toxicity screening [14].

## Method details

### Sample preparation: from cell cultures to propionylated histone peptides

After harvesting the cell cultures, histones were extracted as described by Govaert et al. [15] (cf. *Protocol 1* in supplementary material). First, the nuclei were isolated from frozen cell pellets by resuspension in hypotonic lysis buffer (HLB). A number of 10^6^ cells was resuspended in 200  µL of HLB (10 mM Tris–HCl pH 8.0, 1 mM KCl, 1.5 mM MgCl2) supplemented with 1 mM dithiothreitol (DTT), Halt protease, 1 V/V% EDTA-free phosphatase inhibitor cocktail (788,441, Thermo Fisher), and 1 V/V% phosphatase inhibitor cocktails II and III (P5726 and P0044, Sigma-Aldrich). Subsequently, this mixture was incubated for 30  min on a rotator at 4°C after which the nuclei were pelleted, and the supernatant was discarded. The pellet was resuspended in 125  µL of 0.4 N HCl and incubated for 30  min on a rotator at 4°C. Next, the histones were precipitated with 33% trichloroacetic acid on ice for 30  min. A histone-load corresponding to 400,000 cells was quantified by gel-electrophoresis on an 8–16% TGX gel (Biorad) (cf. *Protocol 2* in supplementary material).

Note that histones come in a large number of proteoforms, which we convert into their respective peptidoforms by using a trypsin digest after chemical derivatization by propionylation [16,17]. The purpose of this propionylation reaction is twofold. Firstly, histone proteins consist of high levels of lysines (K) and arginines (R) compared to other proteins. This has consequences for the protein digestion, the process by which proteins are broken down into smaller peptides. In proteomics, the commonly used enzyme is trypsin, which specifically cleaves behind K and R. However, the use of trypsin for digestion of histone proteins results in relatively short tryptic peptides and would vary based on the modification status of the substrate histones. Therefore, propionlylation is performed as this modifies all free primary amines, resulting in an Arg-C specificity instead of a tryptic specificity [18]. Secondly, short tryptic peptides will show less retention on a C18 column during reversed-phase (RP) high-performance liquid chromatography (HPLC). The presence of a propionyl-group increases the hydrophobicity, thereby enhancing the retention and separation of the peptides during HPLC [18]. The propionylation reaction was performed as previously described [18], [19], [20] (cf. *Protocol 3* in supplementary material). Briefly, the remaining purified histones of each sample were first vacuum dried and then dissolved in 20  µL 1 M triethylammonium bicarbonate (TEAB) buffer, pH 8.5. Next, 20  µL of propionylation reagent (propionic anhydride: 2-propanol 1:80 (v/v)) was added for incubation of 30  min at room temperature (RT). This was followed by adding 20  µL MilliQ water (Merck Millipore) for 30  min at 37°C. Next, histones were digested overnight at 37°C using trypsin (enzyme/histone ratio of 1:20 (m/m)) in 500  mM TEAB, supplemented with calciumchloride (CaCl_2_) and acetronitrile (ACN) to a final concentration of 1.0  mM and 5%, respectively. Subsequently, the derivatization reaction was repeatedly implemented to cap the newly formed peptide N-termini. Non-specific overpropionylation at serine (S), threonine (T) and tyrosine (Y) was reversed by resuspending the vacuum dried sample in 50  µL 0.5 M NH2OH and 15  µL NH4OH at pH 12 for 20  min at RT. Finally, 30  µl of 100% formic acid (FA) was added, and the propionylated histones were vacuum dried for further analysis.

### Data acquisition: from propionylated histone peptides to mass spectra

Among the wider scientific community, data-dependent acquisition (DDA) is the preferred untargeted acquisition strategy, mainly due to the straightforward data analysis [21]. Because data analysis of histone samples is notoriously difficult, DDA was adopted as the acquisition strategy for this workflow. First, the propionylated histone peptides were resuspended in 0.1% FA. Next, a beta-galactosidase (Sciex) solution in 0.1% FA (25 fmol bèta-galactosidase/µL) was added to ensure that an injection volume of 5 µL results in 2 µg histones and 50 fmol beta-galactosidase standard on column. Prior to MS-analysis, peptides were separated with HPLC using a low pH RP gradient on the NanoLC 425 system operating in microflow mode (5 µl/min). A Triart C18 150× 0.3 mm column (YMC) was used at 5 µl/min flow rate (0.1% FA with 3% DMSO) with a 60 min gradient from 3%–45% ACN in 0.1% FA for a total run time of 75 min per sample. The sample list was randomized and interspersed with injections of propionylated bovine histone standards (Roche). For the MS-analysis, a TripleTOF 5600 (Sciex) was used. Each cycle in one full MS1 scan (*m/z* 400–1250) consisted of 250 ms followed by MS2 data-dependent trigger events (*m/z* 65–2000, high sensitivity mode). A maximum of 10 candidate ions (charge state +2 to +5) exceeding 300 cps was monitored per cycle and excluded for 10 s, with an accumulation time of 200 ms and using a rolling collision energy with a spread of 15 V. The cycle time was 2.3 s, in order to obtain 10–12 data points per LC-peak (cf. *Parameter file 1* in supplementary material). The combination of HPLC with tandem mass spectrometry (MS/MS) results in a multitude of MS1 and MS2 spectra i.e. graphs in which the intensity is plotted as a function of the mass-to-charge ratio (*m/z*).

### Data analysis: from mass spectra to relative abundance

Raw data from all runs were imported and all runs in a single experiment were aligned in Progenesis QIP 3.0 (Nonlinear Dynamics, Waters) following feature detection. Features are here defined as precursor *m/z*-values in MS1 scans eluting within a limited time frame and displaying an isotopic envelope resembling that of peptides. Many of these can be expected to be peptidoforms derived from histones. To identify these peptidoforms, a predefined number of MS/MS spectra closest to the elution apex were selected per feature and exported for searches using Mascot (Matrix Science). It should be noted that Mascot is a probabilistic algorithm able to run without a more conventional decoy database. Currently, by lack of a decoy strategy that can cope with complexly modified peptides (e.g. histones), such algorithms are strongly encouraged. To ensure untargeted screening, searches are best performed with a set of 9 PTMs. To determine the best PTM set, the twenty MS/MS spectra closest to the elution apex were selected and merged into a single ∗.mgf file. A standard search without biological modifications was performed using a complete Human Swissprot database (downloaded from Uniprot and supplemented with contaminants from the cRAP database (https://www.thegpm.org/crap/)) to identify the proteins present in the sample (assuming at least a single non-modified peptide can be detected for each protein). From these proteins, a FASTA-database was generated. Next, based on a smaller exported *.mgf file consisting of only the three MS/MS spectra closest to the elution apex per feature, a Mascot-search was performed. In this search, the following parameters were included: 1) mass error tolerances for the precursor ions and its fragment ions were set at 10 ppm and 50 ppm respectively; 2) enzyme specificity was set to Arg-C, allowing for up to one missed cleavage site; 3) variable modifications included acetylation, butyrylation, crotonylation, trimethylation and formylation on K, methylation on R, dimethylation on both K and R, deamidation on asparagine (N), glutamine (Q) and R and oxidation of methionine (M); and 4) N-terminal propionylation and propionylation on K were set as fixed modifications. Database searching was performed against the above mentioned custom-made FASTA-database.

Next, the result files containing all the identified peptidoforms were exported in *.xml-format, after which they were imported back into Progenesis QIP 3.0 to annotate the features from which they were derived. Features that were annotated as peptidoforms derived from histones were also manually validated to resolve isobaric near-coelution. Based on the area under the curve (AUC) of these features, percentages of individual hPTMs, or relative abundances (RA), were calculated by dividing the AUC for each peptidoform containing the considered hPTM by the sum of the AUCs for all observed forms of that peptide [11]. In this way, we are able to report trends in single hPTMs. Reporting these trends is useful when cells are exposed to different concentrations of a compound or when a time-lapse experimental design is used. In doing so, the trends enable prediction of the epigenetic toxicity of the investigated compound. In our pilot-study, we chose to implement a hESC cell line as template to study (early) embryotoxicity (see below: method validation). Nevertheless, depending on the type of cells involved, other forms of toxicity can be focused on (e.g. liver cells or kidney cells for respectively hepato- and nephrotoxicity). Through further optimizations of this workflow (see below), it will evolve to become a high throughput, easily applicable toxicological screening assay, which is, to the best of our knowledge, the first of its kind. As a result, this assay can become part of the standard toxicology toolbox and could subsequently be an important advance for among others the pharmaceutical industry (e.g. discovery of epidrugs), the field of personalized medicines, and the field of environmental toxicity.

Because histone analysis is so complex, it is of utmost importance that the raw data is shared publicly for future reference (see below).

## Method validation

### Valproic acid

Valproic acid (VPA) is a drug mainly known for the treatment of epilepsy and seizures, yet is also prescribed in the context of anxiety, migraine, bipolar, and psychiatric disorders [22]. VPA exhibits various mechanisms of action that have not all been fully elucidated. Nevertheless, the effect would primarily be attributed to an increase in gamma-aminobutyric acid (GABA) levels in the brain and the blocking of voltage-gated sodium, potassium, and calcium channels. Additionally, VPA has recently been identified as a histone deacetylase inhibitor (HDACi). Because of this inhibition, VPA has been presented as a promising antitumor agent as it may potentially enhance the expression of apoptosis-initiating genes, as well as increasing the cytotoxicity of chemotherapy [22]. VPA is currently in trial for the treatment of ovarian cancer [23]. Therefore, this epidrug, with a proven effect on the histone code, is the ideal compound to verify the efficacy of our assay.

### Stepwise overview of the analysis of VPA

The design of this validation experiment can be summarized as follows: hESCs were treated with four different concentrations of VPA (0.04 mM, 0.2 mM, 1 mM, and 5 mM), with each concentration in quadruplicate. The LC-MS/MS runs of the treated hESC-samples were interspersed with bovine histone standards (one in the beginning, one in the middle, and one at the end of the batch). In addition, hESC-samples that were only incubated in H_2_O (without VPA) were added as a control due to the use of H_2_O for dissolving the VPA-samples. The choice of solvent is based on the solubility of the involved compound. For other compounds (e.g. all-trans retinoic acid, methotrexate or trichostatin A) a different solvent such as for instance DMSO may be preferred. Of the H_2_O-samples, four biological replicates were included, as well as technical replicates for these samples.

Sample preparation and data acquisition were performed as described in the section `data analysis' (cf. *Protocol 1–3* and *Parameter file 1*). Focusing on the data analysis, a step-by-step overview is given in the section below. The mass spectrometry proteomics data have been deposited to the ProteomeXchange Consortium via the PRIDE [24] partner repository with the dataset identifier PXD019652 and 10.6019/PXD019652.

**Fig. 3.1:**
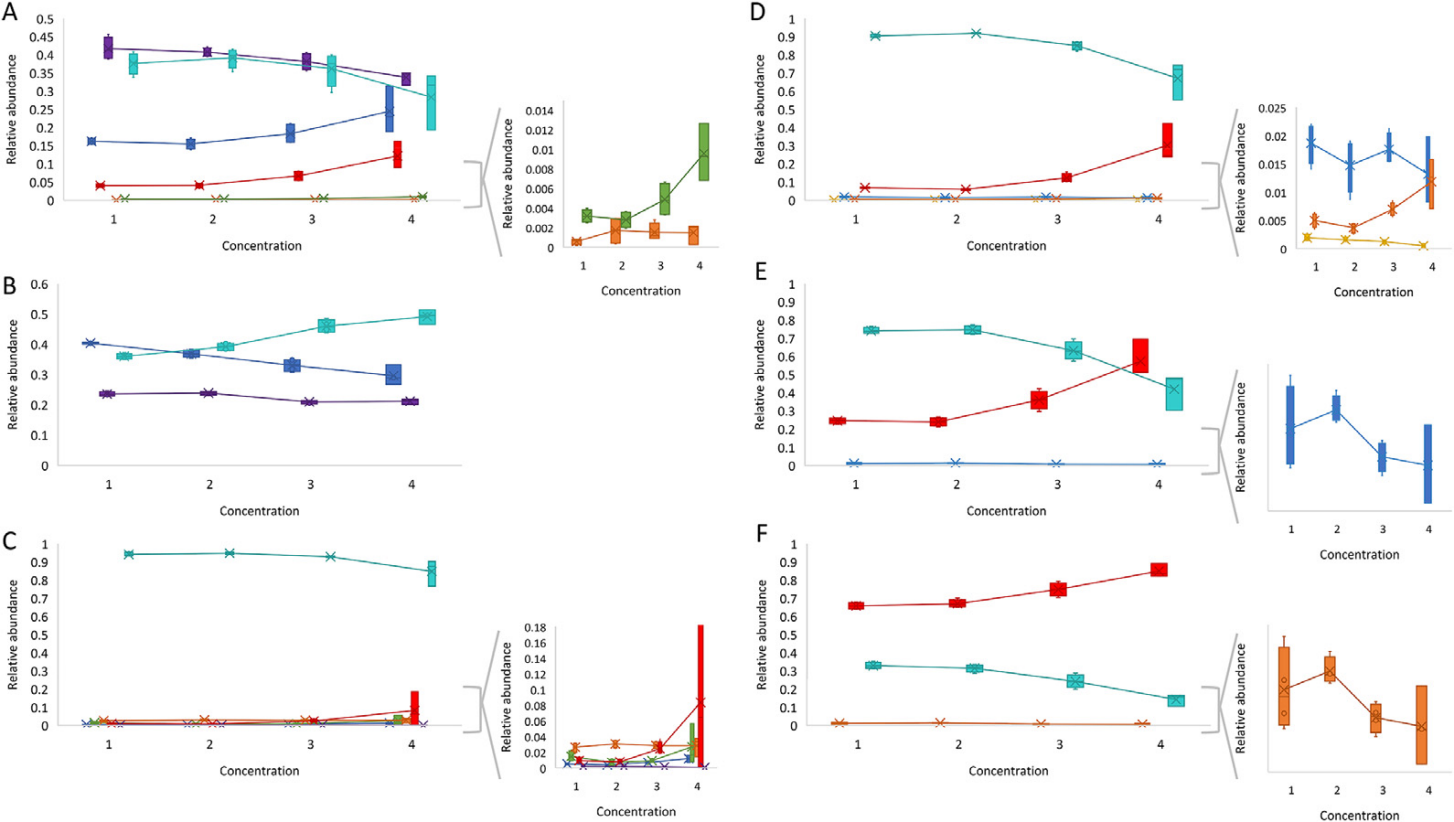
Relative abundance in function of increasing concentrations 1–4 (with 1 = 0.04 mM, 2 = 0.2 mM, 3 = 1 mM, and 4 = 5 mM) of VPA of (A) K27 of histone H3, (B) K36 of histone H3, (C) K5 of histone H4, (D) K8 of histone H4, (E) K12 of histone H4 and (F) K16 of histone H4. The represented hPTMs are acetyl (), formyl (), crotonyl (), trimethyl (), dimethyl (), monomethyl/butyryl (), and the unmodified form ().

1. *.wiff files from the DDA runs of all samples were imported and automatically aligned and peak picked in Progenesis QIP 3.0.
2. Filtering was done eliminating ions with a charge +1.
3. Normalization was verified to ensure adequate loading of each sample.
4. The FASTA-database was generated based on the twenty MS/MS spectra closest to the elution apex per feature as described above. (cf. Error tolerant search - *Parameter file 2* in supplementary material).
5. To verify the presence of beta-galactosidase in the samples and the efficiency of the propionylation reaction, a Mascot-search (cf. Trypsin search) was performed with trypsin as enzyme and no fixed modifications (cf. *Parameter file 3* in supplementary material).
6. A Mascot-search (cf. 9 hPTM search) was performed based on the exported *.mgf file consisting of the three MS/MS spectra closest to the elution apex as described above (cf. *Parameter file 4* in supplementary material). This resulted in 249 identified proteins in Mascot.
7. After importing the identifications (obtained in 6) in Progenesis QIP 3.0, the feature detection of the annotated histone peptides was manually validated and a new *.mgf file (rank 3 again) was exported for a Mascot-search with the same parameters as the search in 6. This resulted in 250 identified proteins of which histone H3, H4, H1, and H2A were the best scoring.
8. The resulting *.xml file was imported in Progenesis QIP 3.0 yielding in 2277 spectrum matches of which 240 peptide ions were identified as histone peptide ions.
9. Subsequently, RA was calculated as described above. Focusing on K27 and K36 of histone H3 and K5, K8, K12, and K16 of histone H4 as represented in Fig. 3.1. At first glance, we can immediately observe an increase in the degree of acetylation as a function of the increasing concentration of VPA (except for B due to the absence of acetylation). As VPA is an HDACi, this matches the expectations.

## Side notes

### Sample preparation

Over the past decade, the sample preparation workflow was already extensively optimized. However, the digestion with trypsin in combination with the propionylation reaction is a time-consuming process. Even more importantly, this also introduces chemical noise that interferes with quantification of important hPTMs [25], [26], [27]. Several biological hPTMs are affected by propionylation such as (i) propionyl groups can be chemical or biological in origin, undermining correct biological interpretation, (ii) methylated Ks are propionylated to butyryl, rendering methyl and butyryl indistinguishable [26], and (iii) propionylation can also introduce formylation, again undermining correct biological interpretation [28]. Unfortunately, no good alternative for chemical derivatization has been proposed to this date for the study of histones*.* Therefore, we are currently investigating digestion with the enzyme gingisrex (GRX, Genovis) that could be promising for the future. This is a novel, highly specific protease that does not cleave after K and thus eliminates the need for propionylation. Consequently, this workflow yields many benefits: (i) a simplified workflow with reduced technical variation [18], [19], [20], (ii) an increased LC-resolution for similarly modified peptidoforms, thus reducing ambiguity [20,29], (iii) less complex peptides, allowing better predictions, and (iv) a reduced chemical noise [25], [26], [27].

### Data acquisition

Currently, the most widespread acquisition strategy to investigate proteins in an untargeted way is DDA, especially given its convenience of data analysis. However, the use of DDA for histones has several limitations, both in terms of identification and quantification [21]. To overcome these limitations, data-independent acquisition (DIA) has emerged as an acquisition strategy with considerable potential [21,30]. Sequential window acquisition of all theoretical fragment ion spectra (SWATH) is such a DIA-approach that holds great promises for the study of histones [31]. In 2019, we uncovered SWATH's full potential to study histones in an untargeted way (hSWATH) [20]. However, to implement hSWATH in the workflow and to enable the assay on a large (industrial) scale, the sample throughput during the acquisition needs to be increased. To accomplish this, the aim is to reduce the length of the LC -gradient while optimizing the window size of the hSWATH approach. In time, we aim to translate the most common histone targets into a multiple reaction monitoring (MRM) assay.

### Data analysis

As an alternative to the data analysis workflow described above, sequential searches can also be incorporated [10,32]. This established method, described by Willems et al. [32], allows an untargeted screening of all relevant hPTMs without ambiguity of annotation. However, in order to ensure a user-friendly assay, we have opted not to incorporate this in the workflow. Finally, RA is currently reported as a biologically interpretable concept, but this approach suffers from several limitations, most importantly the fact that it become more accurate as more hPTMs are identified on the same peptide stretch. Therefore, efforts are still being made to improve the reporting in such a way that the generated results of the assay are as comprehensible as possible.

### Alternative approaches

Traditionally, the analysis of histone modifications was being performed using antibody-based techniques (e.g. western blotting, immunofluorescence and chromatin immunoprecipitation), which are site-specific. However, these approaches are notoriously expensive and rather laborious. In addition, they are constrained by (i) the restricted number of targets under investigation in a single experiment, (ii) antibody cross-reactivity and epitope occlusion, which results in a lack of specificity, and (iii) the absence of combinatorial information [33,34]. As an alternative, different MS-based workflows have been presented, each of which with its own advantages and limitations. For example, using top-down MS (i.e. without digesting the samples first) is a very promising approach to retain all the combinatorial information over the histone backbone. However, histones can theoretically generate 7 · 10^17^ different proteoforms when considering all the possible hPTM combinations both at the level of acquisition and data analysis, this approach has not yet matured to a level that allows large-scale throughput. Other bottom-up approaches like SILAC allow for more accurate quantification, but are expensive and cannot be applied to certain cell types like hESCs, because these cells convert the heavy amino acids into other amino acids [35].

## Declaration of Competing Interest

The authors declare that they have no known competing financial interests or personal relationships that could have appeared to influence the work reported in this paper.

## Appendix Supplementary materials

Image, application 1
