# Supplementary material for: Comprehensive histone epigenetics: A mass spectrometry based screening assay to measure epigenetic toxicity
Source: MethodsX. 2020 Sep 5;7:101055. doi: 10.1016/j.mex.2020.101055 (PMC7508989; doi:10.1016/j.mex.2020.101055)
Supplement: Supplementary file 1 [file mmc1.docx]

**Supplementary material**

Protocol 1: Histone extraction (HLB)

- Resuspend the cells in cold Phosphate Buffered Saline (PBS): 1*10^6^ cells/50µl.
- Distribute in 1.5 mL Eppendorfs: 200µl = 4*10^6^ cells
- Spin down for 10minutes at 4°C and 300g
- Remove the supernatant
- Add 800 µL Hypotonic Lysis Buffer (HLB) to the cell pellet (200 µL for 1*10^6^ cells)

HLB buffer: 10 mM Tris-HCl pH 8.0, 1 mM KCl, 1.5 mM MgCl2 supplemented with 1 mM DTT, Halt Protease and Phosphatase Inhibitor Cocktail 100x (78440) and phosphatase inhibitor cocktails II and III (P5726 and P0044, Sigma-Aldrich, 1 mL of cocktail for 100 mL of buffer).

| **HLB** | **mM** | **MW** | **10x Solution (g/100ml)** |
| --- | --- | --- | --- |
| Tris | 10 | 121.14 | 1.211 |
| KCl | 1 | 74.55 | 0.075 |
| MgCl2.6H2O | 1,5 | 203.3 | 0.305 |
| DTT | 1 | 154.25 | 0.154 |

| **HLB + Inhibitors** | **1 mL** | **30 mL** |
| --- | --- | --- |
| 10x solution | 100 µL | 3 mL |
| Halt Protease and Phosphatase Inhibitor Cocktail 100x | 10 µl | 300 µL |
| phosphatase inhibitor cocktails II and III | 2 x 10 µL | 2 x 300 µL |
| MilliQ Water | 870 µL | 26,1 mL |
| **Check pH = 8** |  |  |

- Rotate for 30minutes at 4°C to promote lysis of cell membrane (mechanical shear)
- Pellet the nuclei in centrifuge for 10minutes at 4°C and 10 000 g
- Discard supernatant
- Resuspend the pellet in 125 µL (for 1*10^6^ cells) 0.4N HCl by soft pipetting until no clumps left in solution (if necessary: vortex)
- Incubate 30minutes in acid on rotator at 4°C to promote lysis of nuclei and solubilization of histones
- Spin down for 10minutes at 4°C and 16000 g.
- Transfer supernatant to new Eppendorf (histones are present in the acid since they are alkaline proteins)
- Add, drop by drop, TCA until a final concentration of 33% is reached to promote precipitation of histones and invert the tube several times (results in a milky solution)
- Incubate on ice for 30minutes
- Spin for 10minutes at 4°C and 16000 g to pellet the histones
- Remove the supernatant (be careful: the pellet is not always visible)
- Add ice-cold acetone (do not resuspend the pellet) to remove TCA, make sure the pellet is fully covered with acetone
- Spin for 5minutes at 4°C and 16000 g
- Remove the supernatant
- Add cold acetone again (do not resuspend the pellet) to remove TCA.
- Spin for 5minutes at 4°C and 16000 g
- Remove the supernatant
- Dry at room temperature for 30minutes (until no acetone left)
- Resuspend in MilliQ water (50 µl for 1x10^6^ cells)
- Transfer 400.000 cells (20 µl) to a new Eppendorf tube for gel-electrophoresis (optionally)

(If there are still clumps left: Spin for 10minutes at 4°C and 16000 g and transfer the supernatant in a fresh Eppendorf)

- Vacuum dry the samples (centrivap)

Protocol 2: Gel-electrophoresis

Sample preparation

- - Dry sample (equal to 400.000 cells)
  - Resuspend samples in 10µl laemmli-buffer
  - Add 1µl β-mercaptoethanol in a fume hood to each sample
  - Vortex and spin down
  - Incubate for 7minutes at 95°C in a thermoshaker
  - Spin down

Prepare Criterion Cell

- - Place the criterion cell on ice in a fume hood
  - Remove the sticker from the bottom of the gel cassette and check the gel for cracks
  - Put the gel cassette in the criterion cell
  - Fill the reservoir with running buffer (25mM Tris, 0.1% SDS, and 192mM glycine in MilliQ water) and take out the comb

Running of the samples

- Load the samples and standards (2 µg of bovine histones) on the gel (3 standards per gel: lane 1, lane 9 and lane 18)
- Put the cover on the criterion cell
- Start running the gel on 200V
- Stop running when the frontline is almost gone

Visualization

- Take out the cassette
- Incubate in fixation-solution (7% acetic acid, 10% methanol in MilliQ water) for 10minutes on a shaker
- Wash the gel 3 times for 5minutes in MilliQ water on a shaker
- Incubate in SyproRuby overnight
- Wash the gel 3x for 10minutes in MilliQ water on a shaker
- Visualize the gel by using a Versadoc

Protocol 3: Propionylation and tryptic digestion

Propionylation before

- Vacuum dry the samples (20 µg/sample)
- Add 20 µL TEAB (1M)
- Add 20 µL Prop-reagent (Isopropylalcohol:propionic anhydride (79:1))
- Spin down & Incubate at room temperature for 30minutes
- Add 20 µL H_2_O
- Spin down & Incubate at 37°C for 30minutes
- Vacuum dry samples

Trypsin digest (Final Volume: 50µl)

- Add 500 mM TEAB
- Add CaCl2 and ACN
  - Final conc CaCl2: 1mM
  - Final conc ACN: 5%
- Resuspend trypsin in 500 mM TEAB
- Add trypsin at a 1:20 ratio (w/w) => 1 µg trypsin/20 µg histones
- Spin down & incubate overnight at 37°C
- Vacuum dry samples

Propionylation after

- Vacuum dried samples (20 µg/sample)
- Add 20 µL TEAB (1M)
- Add 20 µL Prop-reagent (Isopropylalcohol:propionic anhydride (79:1))
- Spin down & Incubate at room temperature for 30minutes
- Add 20 µL H_2_O
- Spin down & Incubate at 37°C for 30minutes
- Vacuum dry samples

Reversing overpropionylation hydroxylamine mediated

- Vacuum dried samples (20 µg/sample)
- Add 50 µL 0.5 M NH_2_OH
- Add 15 µL NH_4_OH at pH 12
- Spin down & Incubate at room temperature for 20minutes
- Adjust pH with formic acid: 30 µl 100% FA
- Vacuum dry samples

Parameter file 1: LC-MS/MS

HPLC: low pH RP gradient on a NanoLC 425 system operating in microflow mode

| Precolumn | Triart C18 5 × 0.5mm (YMC) |
| --- | --- |
| Column | Triart C18 150 × 0.3mm (YMC) |
| Flow rate | 5 µL/min (0.1% FA with 3% DMSO) |
| Gradient time | 60minutes |
| Gradient | 3%–45% ACN in 0.1% FA |
| Total run time | 75minutes |

MS: TripleTOF 5600 (Sciex) in high sensitivity mode

| m/z range MS1 scan | 400-1250 m/z |
| --- | --- |
| Accumulation time MS1 scan | 250 ms |
| m/z range MS2 scan | 65-2000 |
| Accumulation time MS2 scan | 200 ms |
| Charge states | +2 to +5 |
| Rolling collision energy | 15 V |
| Cycle time | 2,3 s |

Parameter file 2: Error tolerant search - rank 20 (Mascot)

| Taxonomy | All entries |
| --- | --- |
| Database | HumanMycoCrap_2018_nr |
| Fixed modifications | Propionyl (K) and Propionyl (N-term) |
| Variable modifications | Deamidated (NQ) and Oxidation (M) |
| MS/MS ion search | x |
| Error tolerant search | x |
| Data format | Mascot generic |
| MS/MS tol. ± | 50 ppm |
| Quantitation | None |
| Instrument | ESI-QUAD-TOF |
| Report top | AUTO hits |
| Protein mass | kDa |
| Decoy | N/A |
| Monoisotopic | x |
| Average | N/A |
| Enzyme | Arg-C |
| Max. miss cleavages | 1 |
| Peptide charge | 2+, 3+, and 4+ |
| Peptide tol. ± | 10 ppm |
| # 13 C | 0 |

Parameter file 3: Trypsin search – rank 20 (Mascot)

| Taxonomy | All entries |
| --- | --- |
| Database | HumanMycoCrap_2018_nr |
| Fixed modifications | N/A |
| Variable modifications | Deamidated (NQ) and Oxidation (M) |
| MS/MS ion search | x |
| Error tolerant search | N/A |
| Data format | Mascot generic |
| MS/MS tol. ± | 50 ppm |
| Quantitation | None |
| Instrument | ESI-QUAD-TOF |
| Report top | AUTO hits |
| Protein mass | kDa |
| Decoy | x |
| Monoisotopic | x |
| Average | N/A |
| Enzyme | Trypsin |
| Max. miss cleavages | 1 |
| Peptide charge | 2+, 3+, and 4+ |
| Peptide tol. ± | 10 ppm |
| # 13 C | 0 |

Parameter file 4: 9 hPTM search – rank 3 (Mascot)

| Taxonomy | All entries |
| --- | --- |
| Database | 200331_VPA |
| Fixed modifications | Propionyl (K) and Propionyl (N-term) |
| Variable modifications | [Acetyl (K)](http://fw01c326/mascot/cgi/client.pl?modification;file=..%2Fdata%2F20200327%2FF005338.dat;mod_name=Acetyl%20K%20%28K%29), [Butyryl (K)](http://fw01c326/mascot/cgi/client.pl?modification;file=..%2Fdata%2F20200327%2FF005338.dat;mod_name=Butyryl%20%28K%29), [Crotonyl (K)](http://fw01c326/mascot/cgi/client.pl?modification;file=..%2Fdata%2F20200327%2FF005338.dat;mod_name=Crotonyl%20%28K%29), [Deamidated (NQR)](http://fw01c326/mascot/cgi/client.pl?modification;file=..%2Fdata%2F20200327%2FF005338.dat;mod_name=Deamidated%20NQR%20%28NQR%29), [Dimethyl (KR)](http://fw01c326/mascot/cgi/client.pl?modification;file=..%2Fdata%2F20200327%2FF005338.dat;mod_name=Dimethyl%20KR%20%28KR%29), Formyl (K), Methyl (R), Oxidation (M), Trimethyl (K) |
| MS/MS ion search | x |
| Error tolerant search | N/A |
| Data format | Mascot generic |
| MS/MS tol. ± | 50 ppm |
| Quantitation | None |
| Instrument | ESI-QUAD-TOF |
| Report top | AUTO hits |
| Protein mass | N/A kDa |
| Decoy | N/A |
| Monoisotopic | x |
| Average | N/A |
| Enzyme | Arg-C |
| Max. miss cleavages | 1 |
| Peptide charge | 2+, 3+, and 4+ |
| Peptide tol. ± | 10 ppm |
| # 13 C | 0 |
